# Political leaders and the media. Can we measure political leadership images in newspapers using computer-assisted content analysis?

**DOI:** 10.1007/s11135-015-0242-9

**Published:** 2015-08-06

**Authors:** Loes Aaldering, Rens Vliegenthart

**Affiliations:** 1 Department of Political Science, Amsterdam Institute for Social Sciences Research, University of Amsterdam, Nieuwe Achtergracht 166, 1018 WV Amsterdam, The Netherlands; 2 Department of Communication Science, Amsterdam School of Communication Research, University of Amsterdam, Nieuwe Achtergracht 166, 1018 WV Amsterdam, The Netherlands

**Keywords:** Political leadership images, Political media coverage, Computer-assisted content analysis, Measurement instrument

## Abstract

Despite the large amount of research into both media coverage of politics as well as political leadership, surprisingly little research has been devoted to the ways political leaders are discussed in the media. This paper studies whether computer-aided content analysis can be applied in examining political leadership images in Dutch newspaper articles. It, firstly, provides a conceptualization of political leader character traits that integrates different perspectives in the literature. Moreover, this paper measures twelve political leadership images in media coverage, based on a large-scale computer-assisted content analysis of Dutch media coverage (including almost 150.000 newspaper articles), and systematically tests the quality of the employed measurement instrument by assessing the relationship between the images, the variance in the measurement, the over-time development of images for two party leaders and by comparing the computer results with manual coding. We conclude that the computerized content analysis provides a valid measurement for the leadership images in Dutch newspapers. Moreover, we find that the dimensions political craftsmanship, vigorousness, integrity, communicative performances and consistency are regularly applied in discussing party leaders, but that portrayal of party leaders in terms of responsiveness is almost completely absent in Dutch newspapers.

## Introduction

Political leadership matters. For decades, scholars have examined the topic and most researchers show that political leaders have an impact on voters when they cast a ballot (e.g. Mughan 2000; Bittner 2011).^1^ The often-studied personalization thesis argues, firstly, that the focus (for instance in the media) is changing from parties to party leaders (e.g. Wattenberg 1994; Dalton and McAllister 2000; Karvonen 2010). Secondly, it argues that the content of media coverage on politicians has changed and that more attention is paid to non-political personality characteristics and the private lives of politicians (e.g. King 2002; Kriesi 2012). Thirdly, and most importantly, it argues that party leaders are increasingly important for citizens in their vote choice and, thus, that leader effects have become stronger over the years (e.g. McAllister 2007; Stewart and Clarke 1992; Hayes and McAllister 1997; Wattenberg 1994, 1998).^2^

To study the consequences of political leaders on society, it is a prerequisite to validly measure leadership images. This paper proposes a novel measure for leadership images in media coverage. Political leaders do not operate in a vacuum but in a mediatized environment, where media are citizens’ principal source of political information (e.g. Robinson 1976; Strömbäck 2008). Voters do hardly meet political leaders in real life and form their opinions on leaders mostly based on information in newspapers, on television and on the internet. However, despite the academic interest in political leadership in modern democracies, leadership images in the mass media have received remarkable little systematic attention.^3^ The goal of this study is, first, to develop a measurement instrument for leadership images in the media, based on a large-scale computer-aided content analysis of newspaper articles. To systematically test the quality of this instrument, we assess the relationship between the images, the variance in the measurement, the over-time development of images for two specific party leaders and by comparison with manual coding. Thus, this paper presents not only a computerized measurement instrument for leadership images, but also evidence that it produces valid results in analyzing Dutch newspapers.

In order to develop a valid measurement instrument of leadership images in mass media, it is necessary to decide which leader character dimensions are included. However, in spite of the magnitude of research on the topic of leadership characteristics, the literature is inconclusive not only in the character traits political leaders possess (the political type), but also in the traits that are perceived by and matter to voters (Bittner 2011). The different perspectives on leadership characteristics are insufficiently integrated, which results in the absence of a widely accepted framework or coherent conceptualization of leader character dimensions (e.g. Blondel 1987; Helms 2012). The second aim of this paper is, therefore, to provide a conceptualization of leadership character dimensions that is on the one hand comprehensive enough to integrate the different perspectives in the field and sufficiently extensive to differentiate between different dimensions and on the other hand is parsimonious enough to be feasible in empirical studies. Based on an extensive literature review, we propose an integrated conceptualization of mediatized political leadership characteristics that consists of six character dimensions: political craftsmanship, vigorousness, integrity, responsiveness, communicative performances and consistency. Thus, this study measures twelve leadership images: the positive and negative images on the aforementioned character dimensions.

The paper unfolds as follows. The first section presents an overview of the existing work on political leadership characteristics and provides a detailed description of the proposed new conceptualization of leadership character dimensions. Subsequently, the methods section discusses the data, analytical strategy and the Dutch case. This is followed by the presentation of the descriptive results and analyses that evaluate the quality of the measurement instrument of leadership images in newspapers. Finally, the conclusion summarizes the results and discusses their implications.

## Political leadership images

Leadership characteristics are studied from multiple perspectives, for instance by open-ended or close-ended survey questions that measure voter’s perspective on real or ideal leaders, experts trait evaluation of successful leaders or content analysis of leader’s biographies or speeches. Despite the scholarly attention for leaders’ character traits, current literature is still ambiguous about the amount and nature of the character dimensions that matter for political leaders, which results in the absence of a widely accepted framework or coherent conceptualization of leadership characteristics (e.g. Blondel 1987; Helms 2012).^4^ Although there is disagreement about the number of dimensions and their specific content, most scholars accept the notion that the number of dimensions is limited, usually no more than six (Garzia 2011; Bittner 2011).

There is a large strand in research that suggests that only two dimensions in leaders’ personality characteristics exist, mostly referred to as competence and trustworthiness or competence and character (e.g. Kinder et al. 1980; Popkin et al. 1976; Stewart and Clarke 1992; Greene 2001; Johnston 2002; Bittner 2011). However, a two-dimensional conceptualization is only plausible if the two character dimensions are so broadly defined that in fact multiple character traits fall into one dimension.^5^ We choose not to restrict the conceptualization of leadership characteristics to two character dimensions a priori and provide deductively,^6^ i.e. based on an extensive literature study of the field of leadership characteristics, an integrated conceptualization of leadership character traits, including six dimensions.

In addition, this study focusses on the content of the character dimensions. Current research has not yet resulted in a common understanding of the character traits, which results in conceptual unclarity and leaves room for ambiguous interpretations. This paper contributes to the political leadership literature by providing a conceptualization of six leadership traits that include extensive definitions of the dimensions and clear descriptions of the personality characteristics belonging to them. The six leadership dimensions are political craftsmanship, vigorousness, integrity, communicative performance, consistency and responsiveness. Table 1 presents a schematic overview of how the six leadership dimensions integrate and bring together leadership conceptualizations from previous research.^7^

**Table 1:** Schematic overview of the dimensions in leadership characteristics in scholarly literature

Source	Political craftsmanship	Vigorousness	Integrity	Responsiveness	Communicative performances	Consistency	Other
Borgotta (1964): concept of personality	Intelligence and task interest	Assertiveness			Likeability and emotionality^a^		
Stogdill (1974): scholarly assessment of leadership	Intelligence and task-related characteristics				Personality and social characteristics		Physical appearance and social background
Miller and Miller (1976): voter’s assessment of leaders	Competence	Reliability	Trust		Leadership appeal^b^	Reliability	Personal appearance
Burns (1978): content analysis on biographies of presidents		Transactional leadership^c^			Transformative leadership		
Shabad and Anderson (1979): voter’s assessment of presidential candidates	Specific competence and general competence and reliable^d^	Leadership	Trust		Personality		Background
Kinder etal (1980): Voter’s profile of ideal president	Competence	Competence (as leadership)	Trustworthy				
Bass (1981): scholarly assessment of leadership	Intelligence and Task related characteristics				Energy and personality and social characteristics		Background
Kinder (1980): voter’s assessment of leaders	Competence	Leadership	Integrity		Empathy		
Lord et al. (1984): voter’s assessment of leaders	Competent and educated	Determined and aggressive and decisive	Caring and honest and dedicated	Understanding and outgoing	Verbally skilled and kind		Well-dressed
Glass (1985): voter’s assessment of leaders	Competence	Character	Character		Personal attraction		Personal attraction
Miller et al. (1986): voter’s assessment of presidential candidates	Competence and reliability^e^	Reliability	Integrity		Charisma		Personal comments as background and appearance
Simonton (1986): content analysis on biographies of presidents	Intellectual brilliance and poise and polish^f^	Machiavellianism and achievement drive and forcefulness and pacifism			Moderation and friendliness	Conservatism and inflexibility	Tidiness and physical attractiveness and pettiness and wit
Winter (1987): motive profiles of leaders and followers	Achievement motive^g^	Power motive		Affiliation-intimacy motive	Affiliation-intimacy motive		
Brown etal (1988): voter’s assessment of leaders	Competence and responsibility and political skills	Dynamism^h^	Integrity		Dynamism and empathy and personal style and political skills		Personal style and episodic judgments and social background attributes and political position
Kenney and Rice (1988): voter’s assessment of presidents	Political skill	Strong leadership and ability to lead and administer					Being nervous
Bean and Mughan (1989): voter’s assessment of leaders	Effectiveness and listening to reason and shrewd	Determined and tough and desicive	Caring		Likable as a person	Sticking to principles	
Shanks and Miller (1990): voter’s assessment of presidents	Competence	Leadership	Trustworthiness		Empathy		
Kasperson (Kasperson et al. 1992): key dimensions of social trust	Competence		Commitment and care			Reliability	
Steward and Clarke (1992): voter’s assessment of leaders	Competence			Responsiveness			
Bean (1993): voter’s assessment of leaders	Competence	Strength	Integrity		General likability and harmony		Other personal and policy/party group
Hogan etal (1994): based on big five model of personality	Intelligence and conscientiousness^i^	Surgency and emotional stability	Conscientiousness		Agreeableness and surgency		
Funk (1996): voter’s assessment of leaders	Competence				Warmth		
Bass (1997): assessment of transformative leadership	Intellectual stimulation			Individualized consideration	Charisma and inspirational motivation		
Funk (1999): voter’s assessment of leaders	Leadership effectiveness		Integrity		Empathy		
Pancer etal (1999): voter’s assessment of leaders	Competence		Integrity		Charisma		
Burns and Sorenson (1999): assessment of transformative leadership	Competence	Courage and conviction				Commitment and conviction	
Greene (2001): voter’s assessment of leaders	Competence	Competence	Integrity				
Holmberg and Åkerblom (2001): content analysis of leadership images in media	Pragmatism and procedural^j^	Action-oriented and egalitarianism and consensus	Honesty		Charisma and consensus and modesty		
Johnston (2002): voter’s assessment of leaders	Competence	Competence	Character		Character		
Clarke et al (2004): voter’s perception of leaders	Responsiveness^k^	Competence	Responsiveness		Responsiveness	Competence	
Barisione (2009): theoretical assessment of leaders	Effectiveness	Effectiveness	Trustworthiness		Vision	Trustworthiness	
Adriaansen (2011): voter’s perception of political actor	General competence and problem awareness and promises^l^	Taking charge	Honest and political actors motive	Responsiveness and problem awareness			
Bittner (2011): assessment of use of leadership traits in literature	Competence	(Strength of) leadership	Integrity and character		Empathy and warmth and charisma		
Bittner (2011): assessment of largest internal cohesion	Competence	Competence	Character		Character		

^a^Emotionality is described as ‘tense, gets easily upset, nervous and emotional’

^b^Leadership appeal is described as ‘inspiring, communicative, warm, and likable’

^c^Transactional leadership is described as ‘when one person takes the initiative in making contact with others for the purpose of an exchange of valued things’. Taking the initiative can be considered vigorousness

^d^Reliable is described as ‘hardworking, realistic, pragmatic, careful, capable of handling the job’ and ‘Lazy, impractical, erratic’

^e^Reliability is described as ‘dependable, hardworking and decisive’

^f^Poise and polish is described as ‘sophisticated, formal, mannerly and tactful’. Machiavellianism is described as ‘sly, deceitful, unscrupulous, evasive, shrewd and greedy’. Pacifism is described as ‘peaceable and not courageous’

^g^Achievement motive is described as ‘excellence, moderate risk taking and using feedback’. Affiliation-intimacy motive is described as ‘close relationships with others, interpersonal warmth, self-disclosure and good overall adaption to life’. Power motive is described as ‘concern for impact and prestige, getting formal social power and profligate impulsive actions’

^h^Dynamism is described as ‘strength, decisiveness and charisma’

^i^Conscientiousness is described as ‘hardworking, persevering, organized and responsible’. Surgency is described as ‘sociable, gregarious, assertive and leaderlike’. Emotional stability is described as ‘calm, steady, cool and self-confident’

^j^Procedural is described as ‘coordinators, organizers, planners, long-term oriented, carful and risk-avoiding’. Egalitarianism is described as ‘fair and equal treatment to other, work for equality, delegate and non-authoritarian’. Consensus is described as ‘willing to compromise, being empathetic, humane, good listeners’. Modesty is described as ‘unpretentious, informal, open, humble, low profile and humorous’

^k^Responsiveness is describes as ‘caring, listens to reason and not arrogant’ and competence is describes as ‘capable of strong leadership, desicive, keeps promises and sticks to principles’

^l^Promises is described as ‘promise more than they can deliver’. Political actors motive is described as ‘whether political actors act in the public interest and for the benefit of all the people’

Table 1 shows, first, that nearly all studies include some form of political craftsmanship, most often labeled ‘competence’ (e.g. Johnston 2002; Lord et al. 1984; Shabad and Andersen 1979; Kasperson et al. 1992; Stewart and Clarke 1992). However, other concepts also fit in this dimension, such as ‘intelligence’ (e.g. Borgotta 1964; Hogan et al. 1994; Simonton 1986), task- related skills (e.g. Bass 1981; Stogdill 1974), ‘(leadership) effectiveness’ (e.g. Bean and Mughan 1989; Funk 1999), ‘achievement motive’ (e.g. Winter 1987) or ‘pragmatism and procedural skills’ (e.g. Holmberg and Åkerblom 2001). We define political craftsmanship by the political skills necessary to be effective in the political arena, including intelligence and understanding the rules of the political game. It includes whether a leader is known for possessing sufficient general knowledge as well as knowledge of specific topics and for making well-considered decisions based on comprehensive contemplations. Moreover, political craftsmanship also captures whether the leader is known for his or her political intelligence, implying that the leader understands the game of politics, has insight in the power structures at stake, anticipates strategically on the behavior of colleagues and knows how to influence the debate or decision-making process in its favor. Political experience is an important feature. Thus, the concept we propose is more extensive than only competence or intelligence. There is only one study known that also specifically includes this aspect of political craftsmanship: Kenney and Rice’s (1988) conceptualization includes political skill.

Party leaders who score high on political craftsmanship could be described in the media with qualifications as clever, well-educated, professional, experienced, insightful, strategic or knowledgeable. Negative comments on this dimension are uninformed, thoughtless, ignorant, misjudgment, unwise, inconsiderate and stupid. A recent example of a political leader with a negative image on political craftsmanship is the American president Bush Jr., who was often criticized for his lack of knowledge and (political) intelligence (e.g. Bartels 2002; King 2002).

Second, Table 1 shows that most characterizations also include some form of vigorousness, mostly labeled ‘(strength of) leadership’ (e.g. Bittner 2011; Bean 1993; Kenney and Rice 1988), although other terms are used as well, for instance ‘decisiveness’ (e.g. Lord et al. 1984), ‘assertiveness’(Borgatta 1964), ‘taking charge’ (Kenney et al. 1994; Adriaansen 2011), ‘determination and aggressiveness’ (Lord et al. 1984) or ‘power motive’ (Winter 1987). Vigorous leadership focusses on the leader being described in the media as strong and powerful and whether a leader is portrayed as dominating the decision-making process and making difficult choices when this is necessary. A vigorous leader is as a strong negotiator, decisive and has a powerful and forceful image. Vigorous leaders are discussed in the media as decisive, dominant, courageous, tenacious, persistent and confident. Being discussed as insecure, weak, soft, submissive, docile or a pushover, are examples a non-vigorous leadership image. A striking example of a vigorous leader is Margaret Thatcher. This *Iron Lady* was well known for her hardline and inflexible political style (e.g. Blundell 2008; Evans 1997).

Third, the table shows that the majority of conceptualizations includes some form of integrity, mainly called ‘trust(worthiness)’ (e.g. Shabad and Andersen 1979; Shanks and Miller 1990), integrity (e.g. Bean 1993; Funk 1999; Miller et al. 1986), ‘character’ (e.g. Bittner 2011; Johnston 2002) or ‘caring’(e.g. Bean and Mughan 1989; Lord et al. 1984). However, terms as ‘honest’ (Holmberg and Åkerblom 2001; Adriaansen 2011), ‘conscientiousness’ (Hogan et al. 1994) and ‘dedication’ (Lord et al. 1984) are used as well. More in detail, this concept is described as ‘perceptions that an individual or institution will act in a way that shows concern for and beneficence to trusting individuals’ (Kasperson et al. 1992, p. 170) or by characteristics as ‘moral, (dis)honest, power-hungry, compassionate, decent and care about people like you’ (Greene 2001).

Integrity relates to the supposed intrinsic motivation of political leaders. It includes whether a leader is known for being guided by the needs, wishes and demands of the country, instead of its own. This concerns, thus, whether the political leader has the general interest at heart rather than its personal interest. Comments in the media that stress that the leader is honorable, respectable, honest, decent and uncorrupted exemplify a positive imago on integrity. On the contrary, when a leader is described as a person without integrity, it is emphasized that the leader is deceptive, fraudulent, lying, insincere, depraved, disingenuous or corrupted. One of the most prevalent examples of a political leader who has problems with his integrity image is the American president Nixon, who resigned because of a corruption scandal. Another striking example of a political leader who has a negative image on integrity is the Italian prime-minister Berlusconi, who is involved in multiple law suits for corruption.

The fourth leadership image in this conceptualization is responsiveness,^8^ which is defined as having the capacity of listening to public opinion and knowing the concerns of the electorate. Thus, party leaders with a responsive image are discussed as being accessible, aware of the current problems in society, responsive to the wishes of the public, and approachable. Political leaders with an unresponsive image, on the contrary, are described as being ignorant, arrogant or someone who has lost touch with society or the electorate. Surprisingly few studies include some form of responsiveness in their work. Those who do refer to ‘problem awareness’ (Adriaansen 2011), ‘learning the groups goals’ (Kenney et al. 1994) or ‘understanding and outgoing’ (Lord et al. 1984). Steward and Clarke apply the term ‘responsiveness’, but describe it as ‘affect, caring, good listener, likeable, and trustworthy’ (1992, p. 453), which in our conceptualization actually relates to communicative performances, integrity as well as responsiveness. Clarke et al. (2004) use the term responsiveness, but here it is described as ‘caring, listening to reason and not arrogant’, which in this conceptualization is closer to political craftsmanship, integrity and communicative performances than to responsiveness. It is remarkable that this dimension is mainly overlooked in the literature since, at least in the Dutch case, voters indicate that distrust towards party leaders is for a large part caused by the fact that leaders lost touch with their grassroots (e.g. Adriaansen 2011; Steenvoorden et al. 2009).

Table 1 shows, fifth, that most studies include some form of communicative performances, mainly labeled ‘charisma’ (e.g. Holmberg and Åkerblom 2001; Bittner 2011; Miller et al. 1986) or ‘character’ or ‘personality’ (e.g. Johnston 2002; Bass 1981; Shabad and Andersen 1979; Bittner 2011). General references to the kindness of the leader, such as (general) likability (Bean 1993; Borgatta 1964) or being a nice person (Kenney et al. 1994), references to the empathic capabilities of leaders (Kinder 1983; Shanks and Miller 1990; Funk 1999) or references to transformative leadership (Burns 1978) are also classified as communicative performances. Our definition of communicative performance consists of two parts. First, this leadership image includes whether a political leader is evaluated on its ability to convey its vision on society to the public and, by that, to inspire and mobilize its followers.^9^ In addition, inspiring leaders have the image of being capable of communicating a clear and not to misinterpreted message to the people, thus, being able to unmistakably present their ideas to their electorate. A second, and closely related aspect of the image of leadership communication is the way leaders handle the media. Since voters rarely meet leaders in real life, the media appearance of the leader is often the most direct communication between the leader and its followers (e.g. Van Santen and Van Zoonen 2009; Bos 2012) and therefore of utmost importance in leadership evaluations. Leaders with a mediagenic image are able to leave a positive impression about themselves on media users by their performance and come across as friendly, funny, relaxed, self-controlled, charming or sympathetic. Thus, party leaders with the image of good communicators are discussed in the media as being able to express their vision in a clear way; inspire people with their ideas; and present themselves as empathic, energetic, sympathetic and charming. Party leaders with the opposite image, on the other hand, are described as boring, unpleasant, antipathetic or uninspiring. An example of a political leader with a positive communicative image is the American president Obama. In the 2008 presidential campaign, he inspired enormous amounts of people to vote for him (even notorious non-voters) with slogans as “Yes we can” and “Change we can believe in” (e.g. Thomas 2009).

Finally, the table shows that only a few studies refer to something conceptually close to ‘consistency’. Scholars have described this concept as ‘you know where he stands on issues’ and ‘has a well-defined program for moving the country ahead’; ‘dependable’ (Miller et al. 1986, p. 528); or by comments as ‘the fulfillment of expectations and faith’ and ‘predictability does not necessarily require consistency of behavior (…) more consistency in values’ (Kasperson et al. 1992, p. 170). We define consistency by both stability and reliability. This image includes whether a political leader is described as having opinions and views on society; positions on issues; and corresponding actions that are consistent over time. Of consistent leaders it is known what they stand for, that they will keep their promises and behave in a predictable manner. When leadership is discussed in the media as consistent, it emphasizes that the leader is unchangeable, accountable, foreseeable, dependable or trustworthy. Unreliable leadership, on the contrary, is described as inconsistent, capricious, unpredictable, irregular, erratic or unfaithful. For instance, the Dutch politician Bos is accused of being a flip flopper on the issue of social security by his political opponent, the prime-minister at the time, Balkenende.

The six dimensions of the proposed conceptualization of political leadership characteristics appear to integrate the traits proposed in the literature quite well.^10^ The six dimensions are all theoretically distinguishable, but at the same time not too broadly defined to be inapplicable in empirical research. Moreover, most leadership characteristics found in the literature that are not included in this conceptualization are non-personality traits, such as demographics or physical appearances (e.g. Lord et al. 1984; Miller et al. 1986; Bean 1993). Only in two instances do studies refer to characteristics that do not fit the six dimensions we identified. First, Kenney et al. (1994) introduce the characteristic ‘being nervous’ and describe it as coming into conflict and trying to be accepted. Second, Simonton (1986) introduces the characteristics ‘tidiness’, ‘pettiness’ and ‘wit’ and describes them respectively as ‘methodological, organized, thrifty and not courageous’; ‘greedy and self-pitying’; and ‘humorous, witty, self-confident and cautious’. Since these characteristics seem too narrow in scope to be considered personality dimensions and only one author refers to these characteristics, we believe it is justified to exclude them from our conceptualization without compromising the goal of integration of the field. Therefore, we believe that this conceptualization of leadership images could serve as a comprehensive framework for studying leadership characteristics.

## Methods

The empirical part of this paper draws on a computer-aided content analysis of Dutch newspaper articles from September 2006 till September 2012, including the full campaign periods of three national parliamentary elections (2006, 2010 and 2012). The dictionary-based approach is applied, where the frequency of pre-specified words, belonging to pre-specified categories, are counted. Based on these frequencies, the relative importance or changes over time of the categories in the texts can be determined. Computer-aided content analysis has some major advantages over classical content analysis, such as perfect reliability, low costs, and possibilities for analyzing large amounts of data for considerable periods in time (e.g. Morris 1994; Bligh et al. 2004). Here we rely on a dictionary-based approach, working with pre-defined wordlists to capture the various central concepts. Dictionary-based computerized content analysis also has some drawbacks. Incorrect coding might occur since the context of the text is usually not considered and computer programs are unable to always correctly connect references to the noun they refer to (Morris 1994). Notwithstanding these difficulties, previous research has shown that computer-assisted content analysis based on the dictionary approach can produce results of similar quality as classical content analysis, which relies on human coders, for instance when populism (Rooduijn and Pauwels 2011), negative economic news coverage (Hollanders and Vliegenthart 2011) or the tone of news report on political parties and candidates in election campaigns (Young and Soroka 2012) are measured.

This paper measures the occurrence of political leadership images in newspaper articles. For each of these leadership characteristics introduced earlier, two dictionaries are constructed: one that captures positive comments in terms of the dimensions in the media and one that captures negative ones. Thus, the six leadership character dimensions produce twelve leadership images. The operationalization of the images are initially based on their theoretical definitions and common categorizations in the Dutch thesaurus *Het Juiste Woord* (Brouwers and Claes 1988). We constructed dictionaries that include words that are used for describing party leaders in terms of the images, including both the positive references as well as the negation of negative references for the positive traits and negative references as well as the negation of positive references for the negative traits. The dictionaries, then, where systematically tested and refined by identifying ambiguous words using the ‘keyword in context’ approach, where we studied the word-combinations or phrases in which these words occurred (McTavish and Pirro 1990). Moreover, we identified news articles that contain many evaluative phrases and checked which often-used references were still missing in the dictionaries (for an example of a dictionary, see Appendix).^11^

To subtract the leadership images in newspaper articles, we combined the dictionaries of the images with reference to political leaders. For instance, we searched for newspaper articles that contained at least one of the words of the dictionary that measures positive comments on integrity (such as ‘honest, reliable or integer’, but also ‘not dishonest or not unreliable’ etc.) with a distance of five words to a certain political leader. For each political leader, we applied thirteen searches: the positive and negative dictionaries for the six leadership character dimensions and one search for only the leader’s name. The latter search is necessary to construct a measurement of the occurrence of leadership images in the media relative to leader’s visibility in the media.

All news reports within the period September 2006 till September 2012 that contain at least one reference to a political leader where collected through the digital archive LexisNexis. Political leadership is operationalized by party leaders during campaign periods; chairmen of the party in Parliament (for opposition parties during routine times); and chairman of the party in Parliament or a (prime-) minister (for coalition parties during routine times). We included all political parties with at least one elected chair in Parliament in the time frame under study,^12^ which resulted in 21 political leaders of 11 different parties. Newspaper articles from the national newspapers *de Volkskrant, NRC Handelsblad, NRC Next, Telegraaf, Algemeen Dagblad, Nederlands Dagblad, Reformatorisch Dagblad, Financieele Dagblad, Parool, Trouw,* and free newspapers *Spits, Metro, de Pers* and *DAG* were part of the population.

In total, we found 257.901 references to party leaders (in 144.100 newspaper articles), of which 32.693 included at least one of the twelve leadership images (in 22.343 newspaper articles). We conduct various analyses with different levels of aggregation. At the lowest level (level 1), the unit of analysis is party leader by newspaper article and has 27.510 observations. The second level is aggregated and the unit of analysis is party leader by week, with 3.790 observations. For the third level, the unit of analysis is party leader by month and has 1.206 observations. The leadership images in level 2 and 3 are measured relative to leadership visibility in the media, thus constitute the proportion of articles in which the party leader is portrayed in terms of the leadership images of the total amount of articles in newspapers in which a party leader is mentioned. Lastly, the level of analysis in level 4 is the party leader and has 21 observations.

There are multiple way to systematically assess the quality of the measurement instrument, including studying the variation between cases (Gerring 2001, pp. 183–192), the relationship between categories and the reliability and validity of the measurement (Adcock and Collier 2001; Bryman 2008, pp. 137–163). To assess the quality of the computer-aided content analysis, we employ four different criteria. First, we assess whether the six theoretically distinctive leadership images are also empirically distinguishable and, thus, whether they really differentiate between multiple dimensions of political leadership and whether reduction of the number of dimensions is feasible. We test this by analyzing the association between the leadership images by means of correlational analysis, reliability analysis and factor analysis. Second, we look at the variance in leadership images by party leaders, time, campaign periods and media outlets. When the leadership images truly measure the tone in the media on leaders’ character traits, we expect to find significant variation in its use across those various categorizations. Third, we assess the face validity of the measurement in two case studies, by comparing the development of leadership images in the media of two party leaders of which it is known that its public images changed or real-life events have affected its public image in the time span under study. Fourth, to test the validity of the measurement, we compare the computerized content analysis to manual content analysis of a sample of the material. We present the percentage agreement, the standardized Lotus coefficients and the correlations between the two content analyses—giving a numeric indication of the quality of our measurement instrument. The combination of the four criteria provide a convincing evaluation of the quality of the measurement.

The Netherlands between 2006 and 2012 make for an ideal case for this research. The multiparty political system contains many competing parties and party leaders, and in the time span under study there are a substantial number of party leader changes within parties. Thus, there is enough variation on the party leader level. Furthermore, the Netherlands has a pluralistic media environment with relatively high levels of newspaper readership making the media analyses particularly relevant.

## Descriptive results

**Fig. 1:**
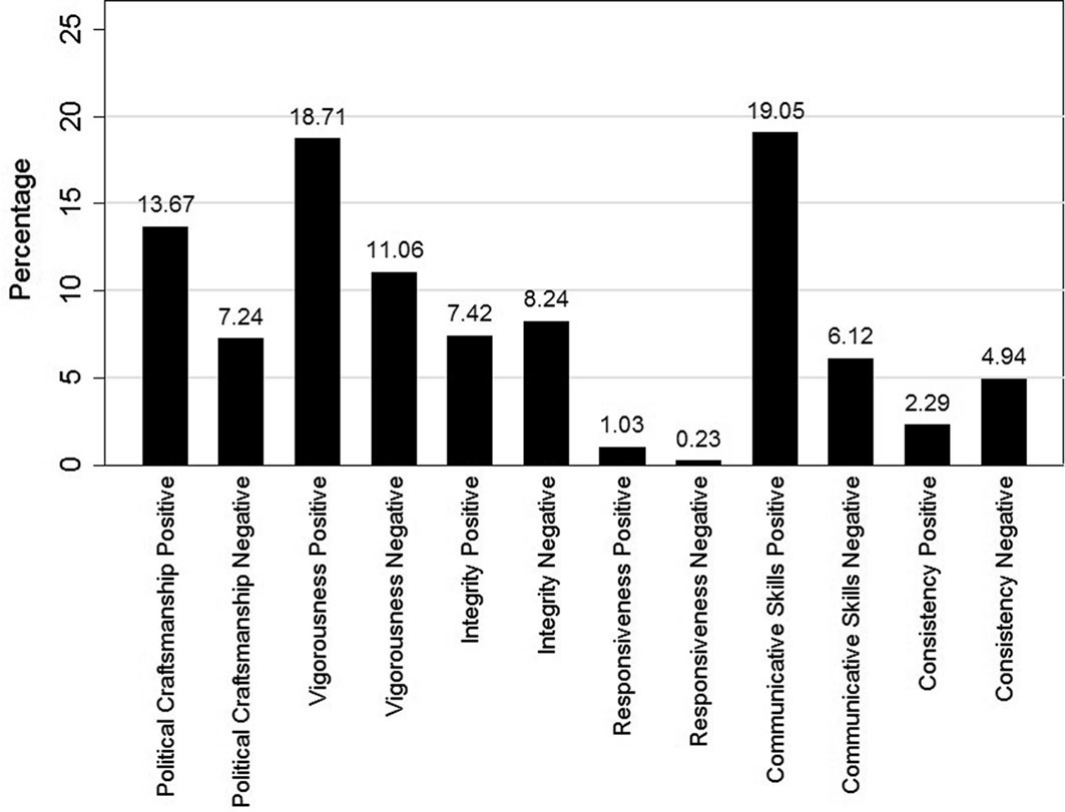
Leadership images in media reports. The figure shows the percentage of the total of images in news media reports that is dedicated to each specific leadership image

Figure 1 shows the distribution of leadership images in newspaper articles. When party leaders are discussed in terms of their character traits, they are most often described with positive comments on communicative performances, closely followed by positive comments on vigorousness and political craftsmanship. Figure 1 shows that, in general, the number of positive images exceeds the amount of negative images, with an exception of integrity and consistency.^13^ Thus, leadership images in media reports are predominantly positive in nature. Additionally, Fig. 1 shows that the responsiveness dimension hardly ever occurs in Dutch newspapers. The positive images on responsiveness form only 1.03 % of the 32.693 leadership images that were found, while the negative image on responsiveness only accounts for 0.23 %. These results could either indicate that our measurement instrument is not able to pick up comments on the responsiveness of party leaders or that not all six, but only five core dimensions of political leadership images are empirically present in Dutch political news coverage.

**Fig. 2:**
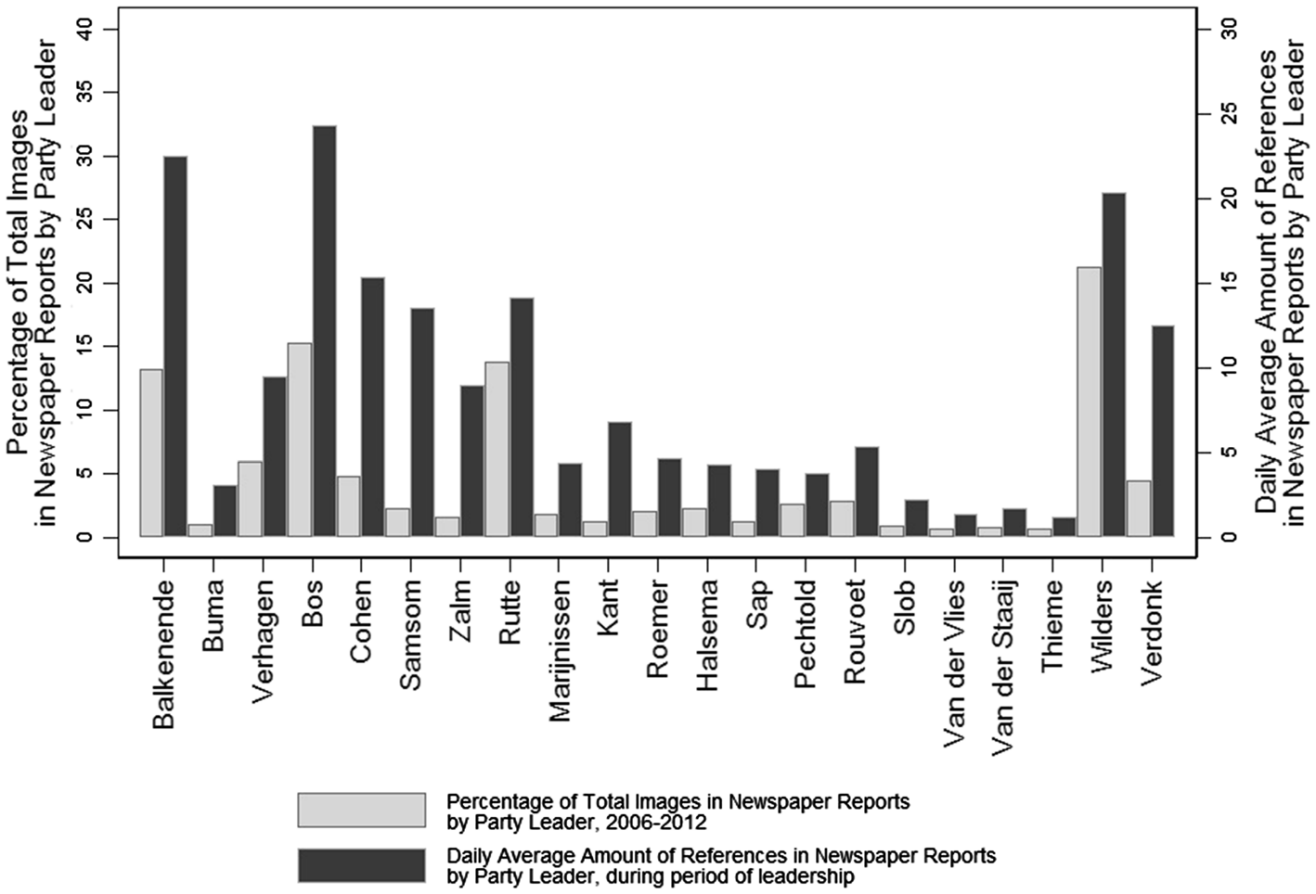
Leadership images and leadership visibility in media report by party leader. The figure shows the percentage of the total of images in news media reports by party leader over the whole period under study (*grey bars*) and the daily average amount of newspaper articles including each party leader, during period of leadership (*black bars*)

Figure 2 shows, first, the distribution of the total of leadership images in media reports over party leaders, for the whole time period under study, and, second, the average amount of daily newspaper articles with references to the party leader during their time as party leader. In absolute terms is Geert Wilders, by far, the most discussed party leader in the Netherlands in terms of these six dimensions, probably because of his exceptional position as leader of the only right-wing populist party in the Dutch electoral system and his extraordinary political style. The number of images in political news for the other party leaders seems mainly to be distributed based on party size. Notable exception is Verdonk, who is discussed in terms of these images relatively often while her party (*TON*) never gained elected seats in parliament. The labour party *PvdA* is the most evaluated party (22.23 % of the leadership images), followed by Wilders’ *PVV* (21.22 %), the Christian Democrats *CDA* (20.04 %), and the liberals *VVD* (15.27 %). The figure shows furthermore, that in terms of visibility, Bos (PvdA), Balkenende (CDA) and Wilders (PVV) score highest. There were, respectively, 24.30, 22.24 and 20.29 newspaper articles per day including their name during the period they where party leader. Visibility seems to depend largely on party size, although there are substantial differences in visibility within parties—for example between Bos, Cohen and Samsom, all three leaders of the *PvdA*.

Additionally, Table 2 shows the absolute amount of references to the party leader (total visibility), the absolute amount of references to the party leader including one of the leadership images (total images) and the percentage of references to party leaders including each image. Relative to the other party leaders, Roemer (socialist party *SP*) scores highest on communicative skills, since 4.32 % of the newspaper articles in which he is mentioned, includes a positive reference to this leadership image. Furthermore, the table shows that Samsom (PvdA) scores higest and lowest on political craftsmanship and vigorousness and highest on integrity. These relatively high scores for Samsom might be influenced because he was only party leader for the short time preceding the election of 2012, while in the campaign periods all leaders receive more attention than usual. Rutte (VVD) scores lowest on communicative performances, while Wilders is relatively most often negatively associated with integrity. Additionally, Bos (PvdA) scores lowest on consistency, while Buma (CDA) scores highest on consitency.

**Table 2:** Leadership Images in Media Reports by Party Leader, relative to time period as Party Leader

Leader	Balkenende	Buma	Verhagen	Bos	Cohen	Samsom	Zalm	Rutte	Marijnissen	Kant	Roemer
Political craftsmanship positive	1.48	1.44	1.71	1.79	2.07	**3**.**17**	2.45	1.80	1.46	1.37	2.01
Political craftsmanship negative	0.79	0.65	0.51	1.06	1.23	**1**.**40**	1.16	1.09	0.49	0.78	0.97
Vigorousness positive	2.15	2.19	2.37	2.29	2.21	**3**.**33**	1.87	2.78	2.40	1.44	2.10
Vigorousness negative	1.28	1.91	1.07	1.58	1.81	**1**.**98**	1.61	1.48	1.15	1.35	1.65
Integrity positive	0.89	2.14	0.55	0.95	1.61	**2**.**51**	0.39	1.04	0.83	0.38	1.06
Integrity negative	1.03	1.02	0.67	0.97	0.95	1.36	0.65	1.03	1.39	0.45	1.21
Responsiveness positive	**0**.**23**	0.09	0.13	0.07	0.12	0.16	0.00	0.14	0.17	0.09	0.07
Responsiveness negative	0.02	0.00	0.02	0.04	0.04	0.00	0.00	0.01	0.00	0.05	0.00
Communicative performances positive	2.22	2.05	2.08	2.24	3.35	4.08	1.55	2.91	2.96	2.46	**4**.**32**
Communicative performances negative	0.72	0.74	0.80	0.77	1.01	0.58	0.65	**1**.**16**	0.59	0.73	0.69
Consistency Positive	0.37	**0**.**47**	0.24	0.39	0.35	0.25	0.13	0.35	0.17	0.17	0.19
Consistency negative	0.65	0.84	0.46	**1**.**15**	0.65	0.54	0.19	0.66	0.24	0.45	0.50
Total images	3.992	291	582	4.304	1.570	470	165	4.481	341	411	625
Total visibility	37.552	2.985	18.449	39.581	10.197	4.644	4.867	31.015	4.786	4.224	4.857

Leader	Halsema	Sap	Rouvoet	Slob	Pechtold	Van der Vlies	Van der Staaij	Thieme	Wilders	Verdonk
Political craftsmanship positive	1.37	2.08	1.37	1.55	1.46	1.60	1.70	1.31	2.14	1.26
Political craftsmanship negative	0.76	1.06	0.52	0.82	0.59	0.83	0.46	0.60	1.17	0.69
Vigorousness positive	1.80	2.94	1.97	2.19	1.74	1.93	2.29	1.43	2.95	2.70
Vigorousness negative	1.09	1.49	1.01	0.46	1.28	1.33	0.92	0.80	1.79	1.11
Integrity positive	1.24	1.14	0.95	1.10	0.66	1.00	0.98	0.52	1.06	0.60
Integrity negative	0.88	0.78	0.38	0.37	0.59	0.66	0.59	0.64	**1**.**65**	1.50
Responsiveness positive	0.16	0.04	0.10	0.00	0.08	0.11	0.20	0.00	0.16	0.14
Responsiveness negative	0.00	0.00	0.01	0.00	0.01	0.00	0.00	0.00	**0**.**06**	**0**.**06**
Communicative performances positive	2.25	2.70	2.05	2.38	2.35	2.16	3.14	1.63	2.78	2.48
Communicative performances negative	0.73	0.51	0.67	0.82	0.71	0.22	0.26	0.64	0.89	0.81
Consistency Positive	0.18	0.24	0.21	0.09	0.17	0.28	0.33	0.08	0.29	0.33
Consistency negative	0.37	0.59	0.33	0.55	0.52	0.17	0.07	0.44	0.57	0.39
Total images	727	346	873	113	843	186	167	203	6.937	804
Total visibility	6.717	3.354	9.496	3.178	8.281	2.038	2.376	2.514	44.703	12.087

Cell entries of the images are the percentages of the total amount of references in newspaper articles that include reference to the image, during their time as party leader. The highest percentages per image are printed in bold. Total images indicates the absolute total amount of references to the party leader including one of the twelve images, during their time as party leader. Total visibility indicates the absolute amount of references to the party leader in newspaper reports, during their time as party leader

**Fig. 3:**
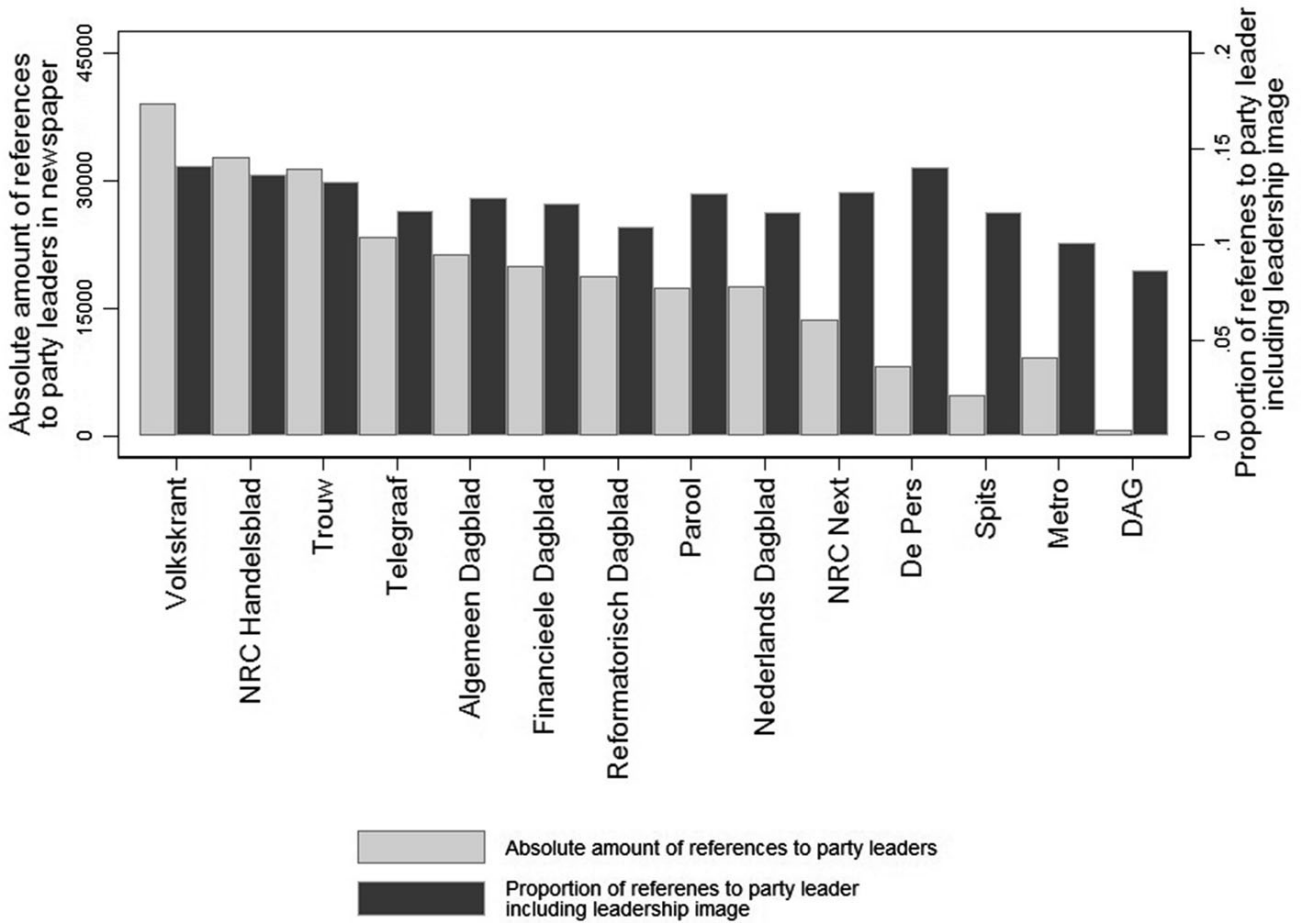
Leadership images in media report by Dutch national newspapers. The figure shows the absolute number of references to party leaders (*grey bars*) and the proportion of references to leadership images thereof by Dutch national newspaper

Figure 3 presents the distribution of leader references and leadership images over Dutch national newspapers. In absolute terms refer the quality newspapers the *Volkskrant* and *NRC Handelsblad* most to party leaders (shown by the grey bars in the figure). The free newspapers (*De Pers, Spits, Metro* and *DAG*) seem to discuss party leaders least. The figure additionally shows the proportion of references to party leaders that includes a leadership image (shown by the black bars in the figure). It shows that most newspapers discuss leadership images in about 11–14 % of the references to party leaders. The largest exception is free newspaper *DAG*, which refers to leadership images in about 8 % of its leadership coverage.

## Distinctiveness of leadership images

A first test of the quality of the measurement instrument of leadership images in Dutch newspapers focuses on whether the twelve theoretically distinctive leadership images are also empirically distinguishable. To this end, we employ correlational analysis, reliability analysis and factor analysis, which provide us with information on the independency of the occurrence of the images in newspapers and whether a reduction of the number of images is feasible. Firstly, Table 3 presents the results of the correlations between the leadership images, aggregated by week and party leader (level 2, relative to media visibility of party leaders) and party leader (level 4).

**Table 3:** Correlations between leadership images in Dutch national newspapers

			Political craftsmanship		Vigorousness		Integrity		Responsiveness		Communicative Performances		Consistency	
			Positive	Negative	Positive	Negative	Positive	Negative	Positive	Negative	Positive	Negative	Positive	Negative
Political craftsmanship	Positive	Level 2	1.00											
		Level 4	1.00											
	Negative	Level 2	−0.05**	1.00										
		Level 4	0.78***	1.00										
Vigorousness	Positive	Level 2	−0.06***	−0.05***	1.00									
		Level 4	0.41	0.54*	1.00									
	Negative	Level 2	−0.08***	−0.02	−0.05***	1.00								
		Level 4	0.63**	0.71***	0.28	1.00								
Integrity	Positive	Level 2	−0.06***	−0.03	−0.07***	−0.02	1.00							
		Level 4	0.41	0.36	0.22	0.55**	1.00							
	Negative	Level 2	−0.03*	−0.04*	−0.05**	−0.04**	−0.01	1.00						
		Level 4	0.25	0.51*	0.71***	0.47*	0.26	1.00						
Responsiveness	Positive	Level 2	−0.01	−0.02	−0.01	−0.03	−0.02	−0.02	1.00					
		Level 4	0.25	0.07	0.40	0.11	0.28	0.34	1.00					
	Negative	Level 2	0.01	−0.02	−0.01	−0.00	−0.00	−0.01	−0.01	1.00				
		Level 4	0.01	0.22	0.23	0.17	−0.21	0.21	0.14	1.00				
Communicative performances	Positive	Level 2	−0.06***	−0.07***	−0.08***	−0.08***	−0.06***	−0.07***	−0.02	−0.02	1.00			
		Level 4	0.37	0.42	0.38	0.44*	0.31	0.45*	0.45*	0.06	1.00			
	Negative	Level 2	−0.07***	−0.03*	−0.03	−0.02	−0.04*	−0.03	−0.01	−0.01	−0.04*	1.00		
		Level 4	0.06	0.40	0.37	0.29	0.13	0.52*	0.26	0.36	0.48	1.00		
Consistency	Positive	Level 2	−0.00	−0.02	−0.04*	−0.03*	−0.02	−0.04*	−0.02	−0.04*	−0.04	−0.03	1.00	
		Level 4	0.16	0.28	0.32	0.51*	0.47*	0.26	0.12	0.40	−0.07	0.19	1.00	
	Negative	Level 2	−0.05**	−0.03*	−0.04*	−0.04*	−0.03	−0.02	−0.13	−0.01	−0.05***	−0.01	−0.02	1.00
		Level 4	0.04	0.21	0.10	0.38	0.24	0.29	−0.15	0.27	0.05	0.37	0.60**	1.00

Cell entries are correlation coefficients between the leadership images. Level 2: unit of analysis is political leader by week (n = 3.790). The leadership images in level 2 are measured relative to leadership visibility in the media. Level 4: unit of analysis is political leader (n = 21)

* Indicates significant at p ≤ 0.05; ** indicates significant at p ≤ 0.01; *** indicates significant at p ≤ 0.001

The correlations on the aggregated week-level, controlled for leadership visibility (level 2) are oftentimes insignificant and limited in size. This indicates that there is no (substantial) association between the occurrence of different leadership images in newspapers. These results show that when a party leader is discussed in terms of a certain leadership image, there is no indication that the party leader is also discussed in terms of another leadership image the same week. For instance, if a party leader’s integrity is questioned and, thus, the party leader receives more negative comments than usual on the integrity dimension that week, we find no difference in the amount of positive images on integrity for that party leader the same week. These results indicate that the twelve theoretically distinctive leadership images can also be empirically distinguished from each other.

The correlations on the aggregated party leader-level, a more stringent test, are even less often significant than the correlations on the second level. However, when significant, these correlations are substantially interpretable. For instance, the correlation of 0.60 between the positive and negative images on consistency indicates that both images are not totally unrelated and that when a party leaders receives positive comments on consistency, he or she also has a higher chance of receiving negative comments on consistency, over the entire period under study. Thus, it shows that there is some association between the occurrence of certain leadership images in newspapers during the period under study, although the strength of these correlations do not seem to imply that reduction of the amount of leadership images is appropriate.^14^

Even though the correlational analyses indicate that the twelve leadership images are empirically distinguishable, we employ additional formal tests to assess whether the number of leadership images can be reduced by combining them. First, Table 6 in Appendix shows the results of a reliability analysis that assesses how well different scales of leadership images perform, aggregated both on newspaper article (level 1) and week (controlled for leadership visibility, level 2). We test the performance of three different scales: one where all twelve leadership images are included (the evaluative news coverage scale); one where all positive images are included (the positive evaluative news coverage scale); and one where all negative images are included (the negative evaluative news coverage scale). The table shows that all three scales perform badly, since none of the Cronbach alpha scores reaches the critical value of 0.70 (Nunnaly 1978). Thus, we conclude that the twelve leadership images cannot reliably be substituted by ‘leadership evaluation in general’, nor could the twelve leadership images be reduced to ‘positive images’ and ‘negative images’.

Second, principal component factor analysis was conducted, of which Table 7 in Appendix presents the results. The low eigenvalues of the factors on both level 1 (aggregated to newspaper article) and level 2 (aggregated by week, controlled for media visibility) indicate that reduction of the amount of dimensions is useless, since they range between 0.51 and 0.01.^15^ When looking at the (rotated) factor loadings, the same conclusion is reached. Each factor is mainly formed by one of the leadership images instead of a combination of images. These results confirm that reduction of the number of leadership images by combining them is not feasible.

Taken together, we conclude that the twelve theoretically distinctive leadership images are also empirically distinguishable from each other. There exists not much substantial association between the leadership images, none of possibly logical combinations of leadership images form reliable scales and factor analysis shows that reduction of the number of leadership images is not possible. The negative comments do not mirror the positive comments on the same image and the images also seem independent from each other. This indicates that the appearances of leadership images in newspapers are not a zero-sum game, in terms of tone and dimensions. This finding is in line with Bean (1993) and Bean and Mughan (1989), who show that leader’s character trait perceptions by voters are not a zero-sum game either. Therefore, we conclude that including all twelve leadership images constitutes a better measurement of leadership images in newspapers than, for instance, a general positive and negative leadership image or the two-dimensional characterization of competence and trustworthiness.

## Variance in leadership images

A second test to determine the quality of the measurement of the computerized content analysis is an assessment of the variance in leadership images. When these twelve images truly measure party leader characteristics, we expect the variance between party leaders to be substantial. Table 4 shows the results of the analyses of variance, where the influence of the most important differentiating variables on variance in leadership images in the media is measured. We included party leaders, newspapers, time and campaign periods^16^ in the analyses. Again, we study the variance on two levels of aggregation: newspaper article (level 1) and week (controlled for leadership visibility, level 2). The results show that when all leadership images are taken together (in Table 4: leadership images in general) leadership images vary significantly over party leaders, newspapers and campaign periods. This indicates that, in general, on which dimensions and how leaders are discussed in the media differs but none of the party leaders’ evaluations increases or decreases linearly over time (no trend).^17^

**Table 4:** Analyses of variances on leadership images in Dutch national newspapers

		Leadership images in general	Political craftsmanship		Vigorousness		Integrity		Responsiveness		Communicative performances		Consistency	
			Positive	Negative	Positive	Negative	Positive	Negative	Positive	Negative	Positive	Negative	Positive	Negative
Model	Level 1	11.02***	1.98	2.92*	12.67***	3.93**	9.64***	12.76***	1.17	1.31	6.89***	0.42	5.75***	28.04***
	Level 2	–	3.45**	2.45*	21.59***	4.14**	2.49*	9.22***	0.77	1.88	15.08***	1.97	2.85*	3.24*
Party	Level 1	7.95**	0.81	1.17	16.44***	0.11	1.86	43.51***	2.52	2.20	6.39*	0.39	19.63***	75.91***
Leader	Level 2	–	4.64*	3.59	58.25***	12.39***	3.62	34.43***	1.15	4.77*	45.43***	5.30*	1.02	3.55
Newspaper	Level 1	6.77**	0.41	0.08	0.13	4.88*	1.89	0.16	1.04	1.12	1.40	0.29	1.60	5.96*
	Level 2	–	0.22	0.44	4.41*	1.93	0.01	0.01	0.02	1.03	1.38	0.31	4.05*	4.68*
Time	Level 1	0.14	6.00*	4.00*	0.06	0.00	1.01	2.66	0.66	0.77	11.40***	0.58	0.39	6.57*
	Level 2	–	8.41**	3.55	0.18	1.70	2.85	0.01	0.74	0.33	12.49***	1.26	1.22	0.09
Campaign	Level 1	27.55***	1.33	5.91*	30.51***	10.92***	31.94***	9.01**	0.76	1.15	7.23**	0.36	0.60	16.34***
Period	Level 2	–	0.74	1.76	23.72***	0.41	3.30	2.25	1.22	1.44	0.92	0.76	5.20*	4.90*

Cell entries are F-values of the analysis of variances on leadership images in Dutch national newspapers. Level 1: unit of analysis is political leader by newspaper article (n = 27.510). Level 2: unit of analysis political leader by week by newspaper (n = 14.303). The leadership images in level 2 are measured relative to leadership visibility in the media. The independent variable *Time* is included in the level 1-analysis as date and in the level 2-analysis as week

* Significant at p ≤ 0.05; ** significant at p ≤ 0.01; *** significant at p ≤ 0.001

However, when we look at the separate models by leadership image, a more detailed picture emerges. It shows that five images on level 1 and six images on level 2 differ significantly across party leaders. The significance of the influence on leadership images is equally whimsical for newspapers, time and campaign periods. These results indicate that some images vary more strongly between party leaders than others (for instance positive comments on vigorousness and negative comments on integrity), while other images vary more strongly over time or between campaign periods and routine times. Finally, Table 4 shows that almost none of the differentiating variables in the model is significant for, both the positive and negative, responsiveness image, both on level 1 and 2 (the only exception is a significant effect of party leaders on level 2 for negative images on responsiveness). This indicates that the variance in the occurrence of party leader’s responsiveness images in Dutch media cannot be attributed to differences between party leaders, newspapers, campaign periods and trends over time, possibly due to the small number of observations on this leadership image.

## Case studies: Cohen and Wilders in the media

A third assessment of the quality of the computer-assisted content analysis is based on face validity. We provide more detailed information about the over time changes in images for two party leaders of which we know that a change in public image has taken place. We investigate whether these changes can be detected in the results of our content analysis.

**Fig. 4:**
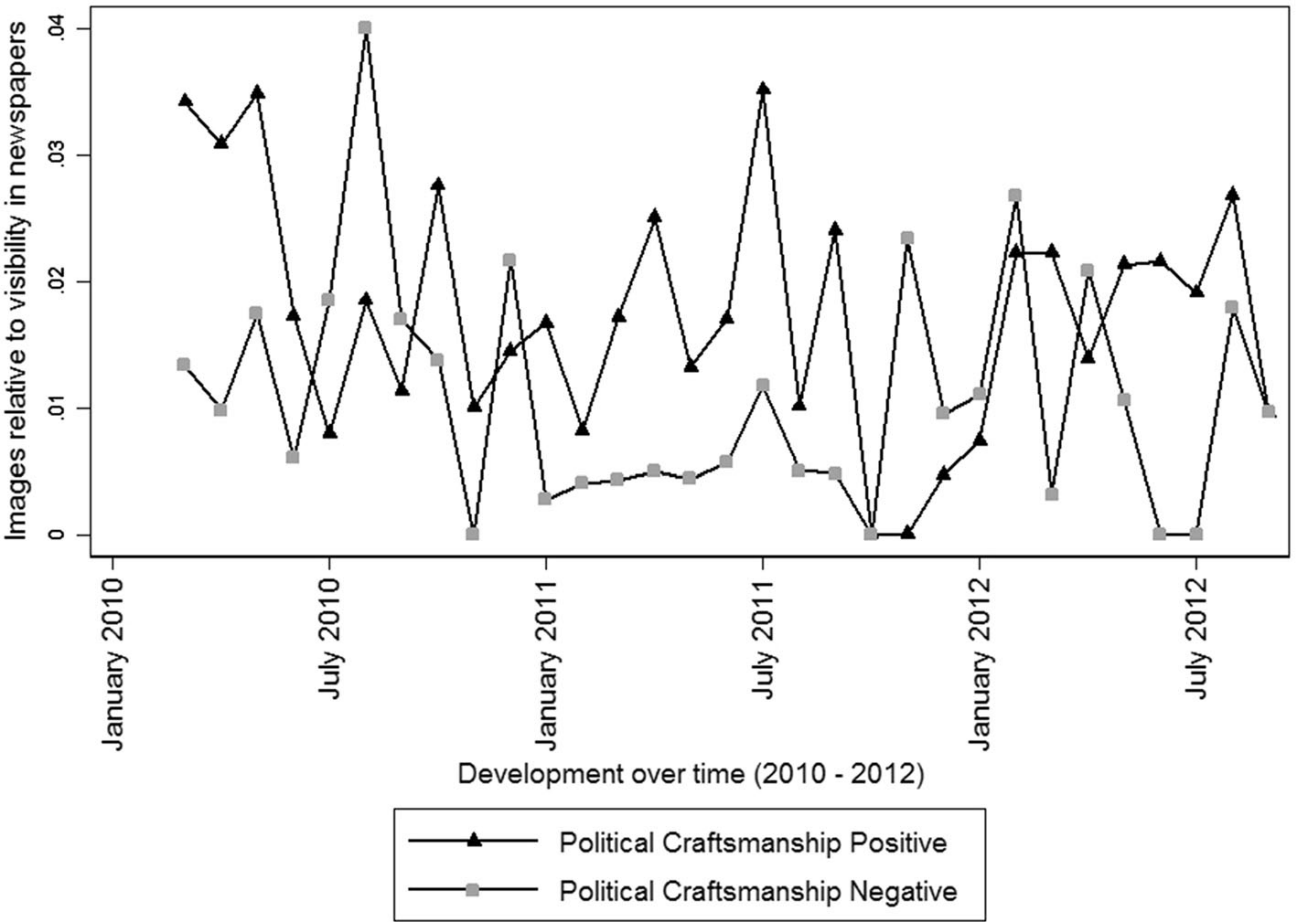
Images of Cohen in newspaper articles. The unit of analysis (level 3) is political leader by month by newspaper (n = 1.433). The leadership images in level 3 are measured relative to leadership visibility in the media

First, we examine more in-depth the news coverage in the media of Job Cohen, party leader of the Labour party (*PvdA*) from March 2010 till February 2012. Figure 4 shows the development of positive and negative comments on Cohens political craftsmanship during this period. When Cohen became party leader of the PvdA, he had built a reputation as mayor of Amsterdam as a very competent and decent administrator (Hendriks 2014). This results in many positive and few negative remarks on his political craftsmanship, reflected in Fig. 4. However, his public image changed after some unsuccessful appearances in the media, where Cohen lacked accurate macro-economic knowledge (Hendriks 2014). In the first seven months as party leader, Cohen failed not only in winning the elections (i.e. PvdA became second-largest party of the Netherlands) but was also not able to get PvdA to become part of government (Goslinga and Turpijn 2011). Cohen’s image shifted in the first months of his party leadership from a capable politician and probable future prime-minister to someone who did not possessed the abilities to successfully lead a party and failed as a member of parliament. This shift in public image is reflected in Fig. 4 where the proportion positive comments on his political craftsmanship sharply drops while the proportion of negative comments on this dimension strongly increases during this period.

**Fig. 5:**
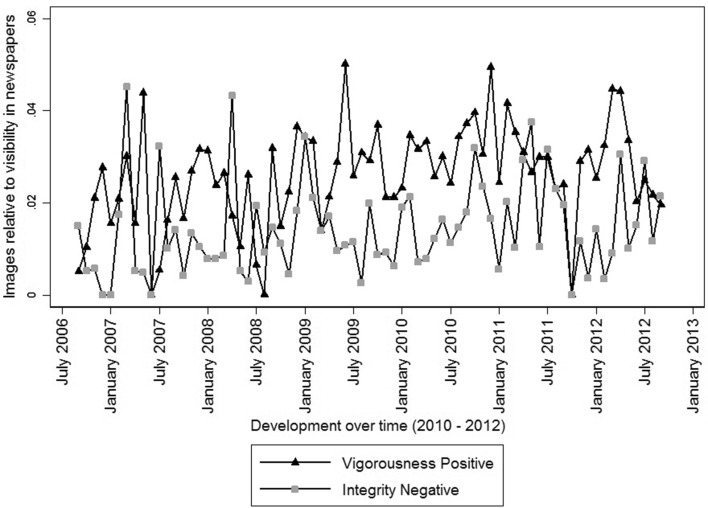
Images of Wilders in newspaper articles. The unit of analysis (level 3) is political leader by month by newspaper (n = 1.433). The leadership images in level 3 are measured relative to leadership visibility in the media

Secondly, we study how Geert Wilders, leader of the right-wing populist party *PVV*, is discussed in the media between 2006 and 2012. Figure 5 shows the development over time of the proportion positive comments on his vigorousness and negative comments in terms of integrity. Wilders is known for his harsh communication style (Bos and Brants 2014), radical ideas, fierce and outspoken criticism on Dutch politics and politicians and his ‘nerve to break taboos’ (Vossen 2011). This picture of Wilders is reflected in the results of the content analysis, since Wilders scores high on positive vigorousness compared to other party leaders. Figure 5 shows, furthermore, peaks in positive comments on vigorousness, for instance, in June 2009, when the European Parliamentary Elections took place in which the PVV became the second-largest party of the Netherlands mainly based on their univocal Eurosceptic position, and in the spring of 2012, constituting the fall of the cabinet after the PVV was unwilling to compromise concerning the reduction of the national fiscal deficit. Additionally, Fig. 5 shows occasional peaks of negative comments on Wilders’ integrity, for instance in March 2007, when he was being accused of hypocrisy when he argued for abolition of dual citizenship while his wife has both the Dutch and the Hungarian nationality, and in March 2008, constituting the release of Wilders’ controversial movie *Fitna* about the evil of the Islam, which caused a lot of commotion in society and for which Wilders was criticized for lying. Lastly, Fig. 5 shows a spike for both positive comments on vigorousness and negative comments on integrity in December 2010/January 2011, constituting the legal trail against Wilders for hate speech and discrimination of Muslims.

These results show that the measurement of leadership traits are in line with trends in media coverage that have been established by previous research. That the distribution of images can be explained by generally acknowledged changes in public images or real-life events suggests that the measurement is useful for analyzing over-time trends.

## Comparison with manual coding

Fourth, we assess the measurement validity of the computer-assisted content analysis by comparing it with manual content analysis. Ten coders were instructed to code the occurrence and tone of the six leadership dimensions for all the party leaders in a stratified random sample of 4055 newspaper articles.^18^ We performed the dictionary-based computerized content analysis on the same 4055 articles, after which the results of both methods are compared. In most instances, when a party leader is mentioned in an article, his or her leadership is not discussed and, thus, none of the leadership images occurs. As a result, the distribution of the occurrence of the separate leadership images relative to leadership visibility in newspapers is highly skewed, which causes difficulties in comparing the two measurements using traditional measures for intercoder reliability. On the one hand, for instance, Krippendorf’s alpha is too conservative, since occasional differences in coding cause a sharp decline in alpha scores (e.g. Schafraad 2009; Lombard et al. 2002). On the other hand, percentage wise agreement might provide a too optimistic picture, since computer and human coder will agree on the large majority of articles scoring negative on the presence of a specific image. To overcome these difficulties, recently, a new measure, Lotus, was developed (Fretwurst 2013). Lotus is an inter coder reliability coefficient that does take into account the distribution of variables (unlike percentage agreement) and assumes that variables with fewer categories provide higher reliability. Thus, the standardized Lotus coefficient controls for chance, but at the same time does not ‘punish’ for dealing with highly skewed distributed dichotomous variables. This makes Lotus the most suitable measure to assess the reliability of our results.

Table 5 presents the results and shows the standardized Lotus coefficients between occurrence of leadership images in the manual and computerized content analysis. Overall, scores are satisfactory, with an average of 0.68, indicating that the computer-assisted content analysis performs well. Additionally, we calculated the percentage agreement and correlations between the observations (aggregated by month, level 3) and find an average percentage agreement between the computerized and the manual content analysis of leadership images of 94 % and an average correlation of 0.56.^19^ Since both the positive and negative comments on responsiveness hardly occur in this sample (only 2.44 % of all manually coded images constitutes a positive image on responsiveness, while only 1.31 % constitutes a negative image on responsiveness), we additionally calculated the reliability scores without the responsiveness images and find an average Lotus coefficient of 0.67, a percentage agreement of 93 % and a correlation coefficient of 0.63 when responsiveness is excluded. These reliability tests indicate that the computer-assisted content analysis performs reasonably well, also when responsiveness is disregarded.

**Table 5:** Comparison of the computer-assisted content analysis and the manual content analysis

		Standardized lotus coefficient	Percentage agreement	Correlation coefficient	Standardized Lotus coefficient	Percentage agreement	Correlation coefficient
		Responsiveness included			Responsiveness excluded		
Political craftsmanship	Positive	0.62	0.91	0.64	0.62	0.91	0.64
	Negative	0.62	0.91	0.61	0.62	0.91	0.61
Vigorousness	Positive	0.63	0.91	0.64	0.63	0.91	0.64
	Negative	0.67	0.93	0.62	0.67	0.93	0.62
Integrity	Positive	0.70	0.94	0.63	0.70	0.94	0.63
	Negative	0.64	0.91	0.70	0.64	0.91	0.70
Responsiveness	Positive	0.78	0.98	0.32			
	Negative	0.81	0.99	0.10			
Communicative performances	Positive	0.62	0.91	0.68	0.62	0.91	0.68
	Negative	0.67	0.93	0.60	0.67	0.93	0.60
Consistency	Positive	0.73	0.96	0.41	0.73	0.96	0.41
	Negative	0.72	0.95	0.77	0.72	0.95	0.77
Average		0.68	0.94	0.56	0.67	0.93	0.63

Cell entries are inter coder reliability scores between the coded leadership images of the computer-assisted content analysis and the manual content analysis. The unit of analysis of the Lotus coefficient and percentage agreement is political leader by newspaper article (n = 8.201 in computer-assisted content analysis/n = 7.883 in manual content analysis). The unit of analysis of the correlation coefficients is the political leader by month (n = 1.206)

## Conclusion

Hardly anyone disputes the general importance of political leaders in modern mediatized societies and the impact of party leaders on voters. However, literature is inconclusive about the amount and nature of the leadership characteristics that matter for political leadership. Moreover, despite the magnitude of research into political leadership, little is known about how party leaders are discussed in the media, which is especially surprising since the media are citizens’ principal source of political information. This paper’s main goal is to develop a measurement instrument that captures party leaders images in the media, based on a large-scale computer-assisted content analysis of Dutch national newspapers, and to systematically test its quality.

However, in order to develop such a measurement instrument, this study first provides a conceptualization of relevant party leader characteristics. A widely accepted framework of leadership character traits is still lacking, since the different perspectives on leadership characteristics are insufficiently integrated. Based on an extensive literature review, this paper presents an integrated conceptualization of leadership characteristics, including six character traits. First, political craftsmanship refers to the skills needed to perform well in the political arena, including general competence, political intelligence and strategic behavior. Second, vigorousness captures strong and powerful leadership, confidence and decisive behavior. Third, integrity refers to leaders’ honesty, corruptness and whether the leader focusses on its own needs or the needs of the electorate. Fourth, responsiveness captures whether the leader is listening to public opinion and knows the concerns of the public. Fifth, we distinguish communicative performances, which refers to both inspiring or visionary leadership and the mediagenic image of the leader, the latter including whether the leader comes across as friendly, clear and charming. Sixth, consistency captures the stability of the visions and actions of leaders and includes whether the leader behaves in a predictable manner.

Based on a large-scale computerized content analysis of newspapers, this study constructed dictionaries that tap into the negative and positive images of party leaders on the aforementioned character dimensions, measuring a total of twelve leadership images. We measured leaders’ images in Dutch national newspapers, including all articles that contain references to one of the party leaders in the period September 1st 2006 till September 12th 2012, which resulted in over 250.000 references to party leaders and almost 33.000 political leadership images in Dutch newspapers. This study, furthermore, systematically assesses the quality of this measurement instrument. First, by means of correlational analysis, reliability analysis and factor analysis, this paper shows that the theoretically distinctive leadership images are also empirically distinctive. Moreover, we provide evidence that these twelve images really differentiate between multiple aspects of leadership characteristics and that a reduction of the number of images is empirically not recommendable. Second, analysis of variance shows that the leadership images vary substantially between party leaders, as expected when the leadership images measure leaders’ characteristics. Third, this paper shows the development of images in the media over time for two politicians and the measurement instruments picks up trends in public images—thus scoring high on face validity. Fourth, we compared the computerized content analysis with manual content analysis of the same leadership images in Dutch newspapers in the same period. The percentage agreements, standardized Lotus coefficients and the correlations all indicate that the computer-assisted content analysis performs sufficiently well. We argue that these four criteria combined convincingly indicate that the computerized measurement instrument for leadership images performs well and produces valid results.

Substantially, this paper additionally shows that party leaders are hardly discussed in terms of their responsiveness to the electorate. Both in the computerized and in the manual content analysis, we find very few positive and negative comments on leaders’ responsiveness. This is remarkable, since the responsiveness of leaders is a very important aspect in the formation of political trust for Dutch voters. A possibly explanation is that voters form their judgements on the responsiveness of leaders based not based on explicit evaluations, but rather on the behavior of those leaders—e.g. whether and how they to news events and which leaders generate media attention on issues that concern the electorate. In that case, responsiveness can be measured in a different manner, for instance by the relative amount of attention leaders spend on issues in their speeches and press reports, instead of the occurrence of leadership images in newspapers. Regardless of the cause, we conclude that the six theoretically distinctive leadership characteristics result in five empirically relevant leadership images when analyzing media content.

Our study is of course not without shortcomings. For one, we restrict ourselves to find only twelve leadership images in newspaper articles. Although the literature does not indicate that additional leadership characteristics are relevant, this deductive approach does not allow us to test whether and how party leaders are discussed in the media on possible other character dimensions. It has been argued that a priori categorizations of words, i.e. constructing dictionaries, must be preferred over categories inferred from the text (e.g. Weber 1983). However, by doing this, we impose our understanding of leadership images on media data and exclude the possibility of finding other leadership images that might occur in newspapers and have not been extensively discussed in previous literature.

Secondly, we only include newspaper articles in our measurement of leadership images in the media. Unlike television coverage, newspapers easily lend themselves for computerized content analysis. However, it might be that leadership images in newspaper coverage differ from images in television news coverage. Future research should address this empirical question.

In conclusion, this research contributes to the existing literature by developing a systematic and integrated conceptualization of leadership characteristics and providing a measurement instrument that validly measures how party leaders are discussed in terms of these characteristics in newspaper coverage. The developed instrument offers the opportunity to answer a range of highly relevant questions. The most pressing one is possibly on the effects of these leadership images in the mass media on citizen’s opinions and political behavior. Then, we cannot only study whether these images in the media affect citizen’s perceptions of party leaders and, subsequently, their voting behavior, but also which voters are more susceptible for which leadership images and under what conditions. This would not only improve our understanding of leadership effects, but also of media effects and voting behavior in general.

## Appendix

### As an example of a dictionary of leadership images in newspapers, we present here the (simplefied) search string for the positive image on the vigorousness dimension

(PARTY LEADER)

with a distance of 5 words from

(daadkracht* or doorzet* or dominant* or zelfvertrouwen* or zelfverzekerd* or moedig* or krachtig* or sterk* or zware beslissing* or slagkracht* or stootkracht* or werklust* or vasthoud* or volhard* or hardnekkig* or onbuigzaam* or onverzettelijk* or verbeten* or durf* or durv* or aanhoud* or doorbijt* or doordouw* or taai* or volhoud* or stellig* or onbeschroom* or dapper* or heldhaftig* or kranig* or onbevreesd* or onverschrok* or stoutmoedig* or ferm* or gedurfd* or gewaagd* or manhaftig* or manmoedig* or kordaat* or geducht* or fier* or stoer* or macht* or robuust* or doortastend* or onderste uit de kan gehaald* or veel uit gehaald or veel mee naar huis genomen* or slagvaardig* or doorzet* or onbezweken* or de boventoon voer* or de broek aan* or de baas* or de staf* or de scepter* or in bedwang* or in het gareel* or kort houd* or onder de duim* or primaat* or oppermacht* or strak houd* or heer en meester* or laten gelden* or onbreek* or ijzer* or onverbreek* or onverbrekelijk* or strenge hand* or harde hand* or rigide* or verzet* or vastberaden* or vastbesloten* or vast bijt* or vast gebeten* or tegendraads* or tegengas* or afzette* or onkwetsbaar* or besluitvaardig* or hoofdrol* or standhoud* or stand houd* or sleutelrol* or hand en tand or ruggegraat* or ruggengraat* or ruggegra* or ruggengra* or “onbetwiste leider” or regent*)

and not with a distance of 5 words from

(geen* or niet* or niks or matig* or weinig* or gebrek aan or ontbreken* or afwezigheid* or verlie* or zonder* or snijden or korten or cao* or voorkeur or bezuinig* or besparing* or doorbroken* doorbre* or inperk* or daling* or dalen* or daal* or zak* or gezak* or groei* or stijg* or verdeeld* or vervuil* or schouder* or verwacht* or “aan de macht” or machtelo* or buiten or “opvallend genoeg” or twijfel* or indruk* or “sterker nog” or fierens or breken* or breek*)

and not (PARTY LEADER with a distance of 5 words from (peiling* or opiniepeiling* or uitslag*))

and not (PARTY LEADER SPECIFICS)

or

(PARTY LEADER)

with a distance of 5 words from

(slap* or bang* or zwak* or gedwee* or nederig* or onderworpen* or volgzaam* or onderdanig* or onopvallend* or gebrekkig* or hulpbehoevend* or krachteloos* or kwetsbaar* or kwetsbare* or lamlendig* or machteloos* or mat or mak or broos or breekbaar* or nietig* or twijfel* or onzeker* or laten piepelen* or laten wegzetten* or besluiteloos* or aarzel* or weifel* or geaarzel* or geweifel* or onbeslist* or buigzaam* or gehoorzaam* or dienstwillig* or gedwee* or gehoorzamen* or achternalopen* or kneedbaar* or meegaand* or onderwerping* or jaknikker* or tegemoet komen* or schikkelijk* or toegeeflijk* or willig* or zwakkeling* or instemmen* or meewerken* or meelope* or overgedienstig* or gedienstig* or figurant* or onverricht* or passief* or toekijken* or inschikkelijk* or lui or traag* or sloom* or gemakzucht* or laks* or ondaadkracht* or onzelfverzekerd* or onvastberaden* or onvastbesloten* or defensief or defensieve or “verslagen indruk” or geterg* or bescheiden*)

with a distance of 5 words from

(geen* or niet* or niks or matig* or weinig* or gebrek aan or ontbreken* or afwezigheid* or verlie* or zonder*)

and not (PARTY LEADER with a distance of 5 words from (peiling* or opiniepeiling* or uitslag*))

and not (PARTY LEADER SPECIFICS)

See Tables 6 and 7.

**Table 6:** Reliability analysis on leadership images in Dutch national newspapers

Scale	Leadership images		Number of images	Cronbach’s alpha	Average inter-item covariance
Evaluative news coverage in general	Political craftsmanship (positive and negative); vigorousness (positive and negative); integrity (positive and negative); responsiveness (positive and negative); communicative performances (positive and negative); consistency (positive and negative)	Level 1	12	0.13	0.0010
		Level 2	12	0.09	0.0000
Positive evaluative news coverage	Political craftsmanship (positive); vigorousness (positive); integrity (positive); responsiveness (positive); communicative performances (positive); consistency (positive)	Level 1	6	0.10	0.0019
		Level 2	6	0.04	0.0000
Negative evaluative news coverage	Political craftsmanship (negative); vigorousness (negative); integrity (negative); responsiveness (negative); communicative performances (negative); consistency (negative)	Level 1	6	0.07	0.0009
		Level 2	6	0.06	0.0000

Reliability analysis based on leadership images in Dutch national newspapers. Level 1: unit of analysis is political leader by newspaper article (n = 27.510). Level 2: unit of analysis political leader by week (n = 3.790). The leadership images in level 2 are measured relative to leadership visibility in the media

**Table 7:** Factor analysis of leadership images in Dutch national newspapers (rotated)

			Factor 1	Factor 2	Factor 3	Factor 4	Factor 5	Factor 6	Factor 7	Factor 8	Factor 9	Factor 10	Factor 11	Factor 12
Political	Positive	Level 1	0.07	0.08	−**0.54**	0.05	−0.02	−0.03	−0.03	−0.03	−0.00	−0.00	–	–
Craftsmanship	Negative	Level 1	0.07	0.08	0.04	0.05	**0.45**	−0.03	−0.02	−0.04	−0.01	−0.01	–	–
Vigorousness	Positive	Level 1	0.09	−**0.62**	0.05	0.04	−0.03	−0.02	−0.03	−0.02	−0.00	−0.00	–	–
	Negative	Level 1	0.09	0.07	0.06	−**0.52**	−0.03	−0.02	−0.03	−0.02	−0.00	−0.00	–	–
Integrity	Positive	Level 1	0.07	0.09	0.07	0.05	−0.03	−0.03	**0.41**	−0.03	−0.00	−0.00	–	–
	Negative	Level 1	0.19	0.18	0.16	0.17	−0.18	−0.19	−0.13	−0.15	−0.10	−0.12	–	–
Responsiveness	Positive	Level 1	0.01	0.02	0.00	0.02	−0.02	−0.00	−0.02	−0.01	**0.23**	−0.01	–	–
	Negative	Level 1	0.02	0.01	0.01	0.02	−0.00	−0.01	−0.00	−0.01	**0.15**	0.01	–	–
Communicative	Positive	Level 1	−**0.62**	0.09	0.05	0.05	−0.03	−0.01	−0.02	−0.02	−0.00	−0.00	–	–
Performances	Negative	Level 1	0.05	0.07	0.07	0.04	−0.03	**0.40**	−0.04	−0.02	−0.01	−0.01	–	–
Consistency	Positive	Level 1	0.03	0.03	0.03	0.02	−0.01	−0.02	−0.01	0.03	−0.01	**0.24**	–	–
	Negative	Level 1	0.09	0.08	0.07	0.06	−0.05	−0.02	−0.04	**0.37**	−0.01	0.01	–	–
Eigenvalue		Level 1	**0.51**	**0.44**	**0.35**	**0.31**	**0.24**	**0.20**	**0.19**	**0.17**	**0.08**	**0.06**	−**0.03**	−**0.54**
Political	Positive	Level 2	0.02	−**0.30**	0.03	−0.01	0.04	0.00	0.01	0.01	0.01	0.00	–	–
Craftsmanship	Negative	Level 2	0.07	0.03	0.04	**0.23**	−0.01	−0.00	0.01	0.02	0.02	0.00	–	–
Vigorousness	Positive	Level 2	0.05	0.03	−**0.29**	−0.02	0.01	0.01	−0.01	0.01	0.01	0.00	–	–
	Negative	Level 2	0.09	0.09	0.03	0.01	−**0.23**	−0.01	−0.01	0.03	0.01	−0.01	–	–
Integrity	Positive	Level 2	0.07	0.07	0.11	−0.04	0.00	−**0.15**	−0.02	−0.05	0.05	−0.02	–	–
	Negative	Level 2	0.08	0.01	0.06	−0.04	0.05	−0.04	−0.04	−**0.18**	0.03	−0.01	–	–
Responsiveness	Positive	Level 2	−0.00	−0.01	−0.03	−0.00	0.04	0.01	−0.01	0.02	0.01	**0.11**	–	–
	Negative	Level 2	0.05	−0.02	0.03	0.06	0.03	−0.00	0.11	−0.01	0.02	0.04	–	–
Communicative	Positive	Level 2	−**0.33**	0.01	0.03	−0.02	0.03	0.01	−0.01	0.01	0.01	0.00	–	–
Performances	Negative	Level 2	0.01	0.13	0.01	−0.05	0.02	**0.15**	−0.03	0.04	−0.01	−0.01	–	–
Consistency	Positive	Level 2	0.02	−0.06	0.03	−0.00	0.03	−0.01	**0.16**	0.06	0.01	−0.01	–	–
	Negative	Level 2	0.06	0.05	0.05	−0.05	0.05	0.04	−0.01	0.03	−**0.16**	−0.01	–	–
Eigenvalue		Level 2	**0.16**	**0.14**	**0.12**	**0.08**	**0.06**	**0.05**	**0.03**	**0.02**	**0.01**	**0.01**	−**0.04**	−**0.36**

Cell entries are the rotated factor loadings of a principal component factor analysis on the leadership images in Dutch national newspapers. Level 1: unit of analysis is political leader by newspaper article (n = 27.510). Level 2: unit of analysis political leader by week (n = 3.790). The leadership images in level 2 are measured relative to leadership visibility in the media. The highest factor loadings are presented in bold

## Footnotes

1 Although some scholars find that the effects of political leadership on election outcomes are non-existent or negligibly small (e.g. King 2002; Bartels 2002).

2 It must be pointed out that the evidence for the personalization thesis is mixed and that scholars also have shown that personalized news coverage did not increase over time (Kaase 1994; Sigelman and Bullock 1991; Vliegenthart et al. 2011) and found that leader effects decreased or did not change in strength over time (e.g. Clarke et al. 2004; Gidengil and Blais 2007; King 2002; Nadeau and Nevitte 2013).

3 Only one study is known that looks specifically at political leadership images in the mass media. By means of an ethnographic semantic analysis, Holmberg and Åkerblom (2001) explore the images of (outstanding) leadership in Swedish media reports.

4 This paper uses leaders’ character traits, leadership characteristics and leaders’ character dimensions interchangeably and, based on psychological research, it covers the magnitude of personality characteristics that are bundled and which are interpreted as one aspect of someone’s character by others (Funk 1996, 1999; Caprara and Zimbardo 2004). Leaders’ character dimensions must be distinguished from leadership images, which refers to the tone on which leaders are discussed in the media. Thus, every character dimension results in two images, a positive and a negative one.

5 For instance, Kinder distinguishes between competence and trust, where he describes competence as ‘not only technically adept (e.g., "knowledge-able," "appoints good advisors"), but also capable of facing hard choices and tackling formidable problems (e.g., "provides strong leadership")’ (1980, pp. 319–320).

6 In contrast to the often applied inductive approach, most often only based on the items that constitute a factor in a factor analysis (e.g. Bass 1981; Simonton 1986; Miller et al. 1986; Borgatta 1964; Miller and Miller 1976).

7 We included studies that introduce an original leadership characterization.

8 Not to be confused with responsiveness as applied in the representation literature (e.g. Stimson et al. Stimson et al. 1995; Soroka and Wlezien 2005; Hakhverdian 2010), where the term is used as measurement of the extent to which the representative changes policy positions based on shifts in public opinion. Although these two meanings of responsiveness are eminently different, they are probably not unrelated. It seems logical to expect that (feelings of) representation depend on the leader’s image of being aware of the wishes of the electorate.

9 Since charisma is still a very diffuse concept without a universal accepted definition (Van der Brug and Mughan 2007), we focus on an inspiring communication style (in line with Kriesi 2012; Wolfsfeld and Sheafer 2006).

10 Although some other challenges arise with this conceptualization of political leadership images. First, some dimensions overlap slightly, for instance vigorousness and political craftsmanship. Second, there is tension between some dimensions, for example, a responsive leader could be difficult to reconcile with a vigorous or consistent leader.

11 All full dictionaries are available upon request.

12 *Trots op Nederland* (TON), the party of former VVD politician Rita Verdonk, was also included although this party failed to obtain a seat in parliament in the National Parliamentary elections of 2010. TON and Verdonk where included because Verdonk, as independent Member of Parliament after she was excluded from the VVD in 2007, was very influential for some time.

13 The reason why the negative images exceeds the positive ones on these two dimensions is probably because these images are most assumed of party leaders which makes it hardly an accomplishment to be integer or consistent.

14 Additionally, we tested correlations between images aggregated on party leader and newspaper article (level 1) and, in line with the correlations on level 2, we found no substantial association between images.

15 Rule of thumb (Kaiser 1960) is that only factors with eigenvalues larger than 1 are useful.

16 Campaign period is determined by the two months preceding the election campaign.

17 Time, here, is included as date. Time in the level-2 analyses is included as weeks. All level-1 models are also estimated with different measurements of time (weeks and months instead of days). The results show an equally whimsical distribution of significance over the separate leadership images and the interpretation of these results is nearly identical.

18 Tests for inter coder reliability between the ten coders of the manual content analysis are based on a random sample of 50 newspaper articles and are satisfactory with standardized Lotus coefficients of 0.86 for the occurrence of the six leadership dimensions (89.33 % agreement between coders) and 0.71 for the tone of the comment (76.25 % agreement).

19 All correlations of coding of leadership images are significant at p ≤ 0.001.
